# Fiber-1 of serotype 4 fowl adenovirus mediates superinfection resistance against serotype 8b fowl adenovirus

**DOI:** 10.3389/fmicb.2022.1086383

**Published:** 2022-12-21

**Authors:** Hao Lu, Yiwen Guo, Zhenqi Xu, Weikang Wang, Mingjun Lian, Tuofan Li, Zhimin Wan, Hongxia Shao, Aijian Qin, Quan Xie, Jianqiang Ye

**Affiliations:** ^1^Key Laboratory of Jiangsu Preventive Veterinary Medicine, Key Laboratory for Avian Preventive Medicine, Ministry of Education, College of Veterinary Medicine, Yangzhou University, Yangzhou, Jiangsu, China; ^2^Jiangsu Co-Innovation Center for Prevention and Control of Important Animal Infectious Diseases and Zoonoses, Yangzhou, Jiangsu, China; ^3^Joint International Research Laboratory of Agriculture and Agri-Product Safety, The Ministry of Education of China, Yangzhou University, Yangzhou, China; ^4^Institutes of Agricultural Science and Technology Development, Yangzhou University, Yangzhou, Jiangsu, China

**Keywords:** FAdV-8b, Fiber-1 of FAdV-4, superinfection, CAR, IBH

## Abstract

In recent years, hepatitis-hydropericardium syndrome (HHS) and inclusion body hepatitis (IBH) caused by serotype 4 fowl adenovirus (FAdV-4) and serotype 8b fowl adenovirus (FAdV-8b), respectively, are widely prevalent in China, causing huge economic losses to the poultry industry. Numerous studies have revealed the mechanism of the infection and pathogenesis of FAdV-4. However, little is known about the mechanism of infection with FAdV-8b. Among the major structural proteins of fowl adenoviruses, fiber is characterized by the ability to recognize and bind to cellular receptors to mediate the infection of host cells. In this study, through superinfection resistance analysis and an interfering assay, we found that Fiber-1 of FAdV-4, rather than hexon, penton, and fiber of FAdV-8b, conferred efficient superinfection resistance against the infection FAdV-8b in LMH cells. Moreover, truncation analysis depicted that the shaft and knob domains of FAdV-4 Fiber-1 were responsible for the inhibition. However, knockout of the coxsackie and adenovirus receptor (CAR) in LMH cells inhibited the replication of FAdV-8b only at early time points, indicating that CAR might not be the key cell receptor for FAdV-8b. Overall, our findings give novel insights into the infection mechanism of FAdV-8b and provide a new target for the prevention and control of both FAdV-4 and FAdV-8b.

## Introduction

Fowl adenoviruses (FAdVs) belong to the family *Adenoviridea* and the genus *Aviadenovirus* (Okuda et al., 2004; Mittal et al., 2014; Niczyporuk, 2016; Benko et al., 2022). FAdVs currently consist of five species (FAdV-A~E) with 12 serotypes (FAdV-1 to 8a and 8b to 11), based on the profile of the restriction enzyme and sera cross-neutralization (Hess, 2000; Kim et al., 2014; Niczyporuk, 2016). FAdVs mainly cause poultry diseases, including hepatitis-hydropericardium syndrome (HHS), inclusion body hepatitis (IBH), and adenoviral gizzard erosion (AGE) (Okuda et al., 2004; Mittal et al., 2014; Niczyporuk, 2016). IBH is generally caused by FAdVs belonging to FAdV-2, FAdV-11, FAdV-8a, and FAdV-8b in 3–5-week-old broilers, and is characterized by mass liver necrosis and the presence of intranuclear inclusion bodies in hepatic cells (Schachner et al., 2018). Besides, HHS and AGE are closely related to the infection of FAdV-4 and FAdV-1, respectively (Mazaheri et al., 1998; Ojkic et al., 2008; Marek et al., 2010; Kaján et al., 2013; Schachner et al., 2016; Garmyn et al., 2018). At present, IBH and HHS caused by FAdV-8b and FAdV-4, respectively, are spread worldwide, threatening the development of the poultry industry (Okuda et al., 2004; Changjing et al., 2016; Ye et al., 2016; Shah et al., 2017; Wang et al., 2018; Chen et al., 2019; Lu et al., 2019). The mechanism of infection and pathogenesis of FAdV-4 has been recently studied, while that of FAdV-8b is not fully understood.

The capsid of FAdV is mainly composed of hexon, penton, and fiber (Russell, 2009; Kulanayake and Tikoo, 2021). Among the three structural proteins of FAdV, fiber protein is the distal part on the surface of the viral particle, and is divided into three domains containing a knob, shaft, and tail, involved in viral attachment and pathogenesis (Tan et al., 2001; Taharaguchi et al., 2012; Shah et al., 2017). FAdVs generally possess two fibers protruding from one penton base, and most FAdVs encode only one *fiber* gene, whereas FAdV-4, together with FAdV-1 and FAdV-10, have two *fiber* genes (*Fiber-1* and *Fiber-2*) (Hess et al., 1995; Marek et al., 2012). Both fibers play vital roles in the infection and pathogenicity of FAdV-4. Fiber-1 directly triggers the infection of FAdV-4, possibly through binding to the CAR (Pan et al., 2020; Wang et al., 2020), whereas Fiber-2 determines the virulence and viral replication of FAdV-4 (Xie et al., 2021; Zhang et al., 2021). Unlike FAdV-4, FAdV-8b has only the *fiber* gene (Ojkic and Nagy, 2000; Gupta et al., 2017). Although fiber-based vaccines can provide protection against the challenge of FAdV-8b (Gupta et al., 2017; De Luca et al., 2020; Lu et al., 2022), the mechanism underlying this protection is not well elucidated. Whether the fiber of FAdV-8b plays a similar role to Fiber-1 or Fiber-2 of FAdV-4 in viral infection and pathogenesis needs to be investigated. In this study, we found that Fiber-1 of FAdV-4, but not hexon, penton, and fiber of FAdV-8b, conferred efficient superinfection resistance against the infection of FAdV-8b in LMH cells, providing evidence for common cellular receptors used by FAdV-4 and FAdV-8b and potential cross-protection between FAdV-4 and FAdV-8b.

## Materials and methods

### Viruses and cells

FAdV-4 strain SD2015 and FAdV-8b strain JSSQ15 were isolated and maintained in our laboratory (Ye et al., 2016). FAdV-7 and leghorn male hepatoma (LMH) cells were purchased from ATCC. FAdV-8a strain AH720 was kindly provided by Prof. Hongjun Chen (Shanghai Veterinary Research Institute). The LMH cells were cultured in F12/DMEM (Gibco, NY, USA) with 10% fetal bovine serum (FBS) (Lonsera, Shanghai, China).

### Plasmids

Plasmids pcDNA3.1-Fiber, pcDNA3.1-Hexon, and pcDNA.1-Penton (fiber, hexon, and penton from FAdV-8b) were constructed and kept in our laboratory. Plasmids pcDNA3.1-F1, pcDNA3.1-F1-Tail-Flag, pcDNA.1-F1-Shaft-Flag, pcDNA3.1-F1-Knob-Flag, pcDNA3.1-F1-Tail+Shaft-Flag, and pcDNA3.1-F1-Shaft+Knob-Flag (F1 was the Fiber-1 of FAdV-4) were stored in our laboratory (Wang et al., 2020).

### Proteins and antibodies

His-Fiber-1 of FAdV-4 and His-Fiber-1 of duck adenovirus 3 (DAdV-3) were generated by the expression in *E. coli* BL2, purified by Ni column, identified by SDS-PAGE, and preserved in our laboratory. The monoclonal antibody (mAb) 1B5 against the hexon of FAdVs was provided by Prof. Hongjun Chen (Shanghai Veterinary Research Institute). The mAb 4D9 against fiber of FAdV-8b, positive chicken sera against FAdV-8b, the mAb 3B5 against Fiber-1 of FAdV-4, and anti-chicken CAR polyclonal antibodies were generated and stored in our laboratory. Anti-Flag mouse mAb (Cat F1804) was purchased from Sigma-Aldrich Co. LLC.

### Superinfection resistance assay

Leghorn male hepatoma cells were transfected with the different recombinant plasmids (pcDNA3.1-Fiber, hexon, and penton of FAdV-8b, pcDNA3.1-Fiber-1 of FAdV-4 and Fiber-1 truncations of FAdV-4). After 24 h, LMH cells transfected with plasmids were infected with the indicated virus at a multiplicity of infection (MOI) of 0.01. At 24, 48, 72, 96, and 120 h post-infection (hpi), the supernatants of infected cells were collected and titrated according to the 50% tissue culture infection dose (TCID_50_), as previously described (Lu et al., 2022).

### An interfering assay using purified His-Fiber-1

Purified His-Fiber-1 of FAdV-4 was first mixed with FAdV-8b at an MOI of 0.01 and subsequently inoculated into the LMH cells. At the same time, purified His-Fiber-1 of DAdV-3 was set as a control. At 2 hpi, the infected LMH cells were washed twice with PBS and then maintained with fresh F12-DMEM containing 1% FBS. At day 5 post-infection, the LMH cells were collected and analyzed by Western blot using mAb 1B5 against the hexon of FAdVs and mAb 4D9 against the fiber of FAdV-8b.

### Indirect immunofluorescence assay (IFA)

Leghorn male hepatoma cells transfected with the plasmids or infected with the viruses were fixed with a prechilled acetone-ethanol (3:2) mixture for 5 min and washed twice with PBS. The fixed cells were incubated with diluted antibodies for 45 min at 37°C. Then, the cells were washed thrice with PBS and incubated with the diluted secondary antibodies (fluorescein isothiocyanate (FITC)-labeled IgG) for another 45 min at 37°C. After washing with PBS thrice, the cells were observed using a fluorescence microscope.

### Western blot

The LMH cells were collected and lysed with lysis buffer (CoWin Biosciences, Taizhou, China) with protease and phosphatase inhibitor cocktail (New Cell and Molecular Biotech, Suzhou, China) on ice for 30 min. After high-speed centrifugation at 4°C, the lysates supernatant was boiled with protein loading buffer. After protein samples were separated by SDS-PAGE, the separated proteins were transferred onto nitrocellulose (NC) membranes. After blocking with blocking buffer (New Cell and Molecular Biotech, Suzhou, China) for 20 min at room temperature (RT), the NC membranes were incubated with the primary antibody diluted in PBST containing 5% skimmed milk at 4°C overnight. After three washes with PBST, the NC membranes were incubated with horseradish peroxidase (HRP)-labeled IgG at RT for 1 h. After another three washes with PBST, the membranes were developed on an ECL substrate and analyzed using an automatic imaging system (Tanon 5200).

### Generation of CAR-KO cell lines by CRISPR/Cas9

The sgRNA against the chicken CAR (XM_416681.5) was designed using the website (www.benchling.com) and then cloned into the lentiCRISPR V2 plasmid. The primers used for sgRNA cloning were as follows: forward primer, 5′-CACCGTGTGTTGCTTCAAACCGAGT-3′ and reverse primer, 5′-AAACACTCGGTTTGAAGCAACACAC-3′. Then, LMH cells were first transfected with gRNA targeting chicken CAR. At 48 h post-transfection, the CAR-KO cell lines were selected using the proper concentration of puromycin, followed by limited dilution, sequencing, and Western blot analysis as previously described (Li et al., 2021).

### Viral growth curves

Leghorn male hepatoma cells in 6-well plates were infected with the indicated virus at an MOI of 0.01. At 2 hpi, the inoculated cells were washed twice with PBS and then maintained with F12-DMEM containing 1% FBS. The cell supernatants collected at 24, 48, 72, 96, and 120 hpi were measured for viral replication kinetics as previously described (Lu et al., 2022).

### Statistical analysis

All data are presented as the mean ± standard deviation. In addition, statistical analysis in this study was performed using a Student's *t*-test or a one-way ANOVA *t*-test using GraphPad 6 software. *p* < 0.05 is considered statistically significant. ^*^, ^**^, ^***^, and ^****^ indicate *p* < 0.05, 0.01, 0.001, and 0.0001, respectively.

## Results

### Fiber, hexon, and penton of FAdV-8b failed to block the infection of FAdV-8b

**Figure 1:**
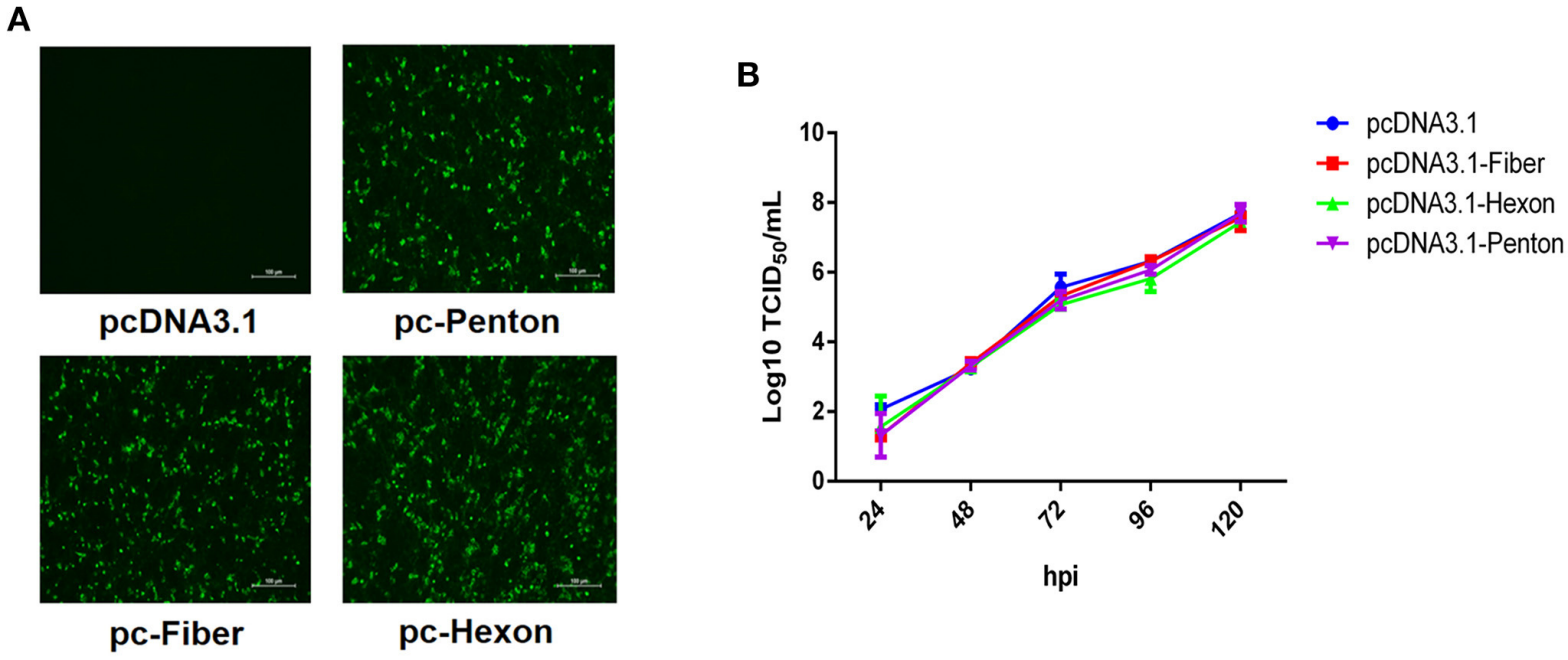
Fiber, hexon, and penton proteins of FAdV-8b were unable to inhibit the infection of FAdV-8b in the superinfection resistance assay. **(A)** LMH cells transfected with pcDNA3.1-Fiber, pcDNA3.1-Hexon, pcDNA3.1-Penton, and pcDNA3.1 were analyzed by IFA using the chicken sera against FAdV-8b. **(B)** Supernatants from the LMH cells infected with FAdV-8b at different time points, which were transfected with pcDNA3.1-Fiber, pcDNA3.1-Hexon, pcDNA3.1-Penton, and pcDNA3.1 and titrated with TCID_50_.

To identify which protein directly mediates the infection of FAdV-8b, we performed a superinfection resistance assay by transfecting pcDNA3.1-Fiber, pcDNA3.1-Hexon, and pcDNA.1-Penton into LMH cells. Similar transfection efficacies and expression levels of fiber, hexon, and penton of FAdV-8b in the transfected LMH cells were verified by IFA (Figure 1A). The superinfection resistance assay revealed that the viral titer of FAdV-8b in the LMH cells transfected with pcDNA3.1-Fiber, pcDNA3.1-Hexon, and pcDNA3.1-Penton presented no difference to that in cells transfected with the control vector pcDNA3.1 at each time point (Figure 1B). Taken together, these data demonstrate that the fiber, hexon, and penton of FAdV-8b could not efficiently block or inhibit the viral replication of FAdV-8b in the susceptible cell line LMH.

### Fiber-1 of FAdV-4 conferred efficient superinfection resistance against FAdV-8b

**Figure 2:**
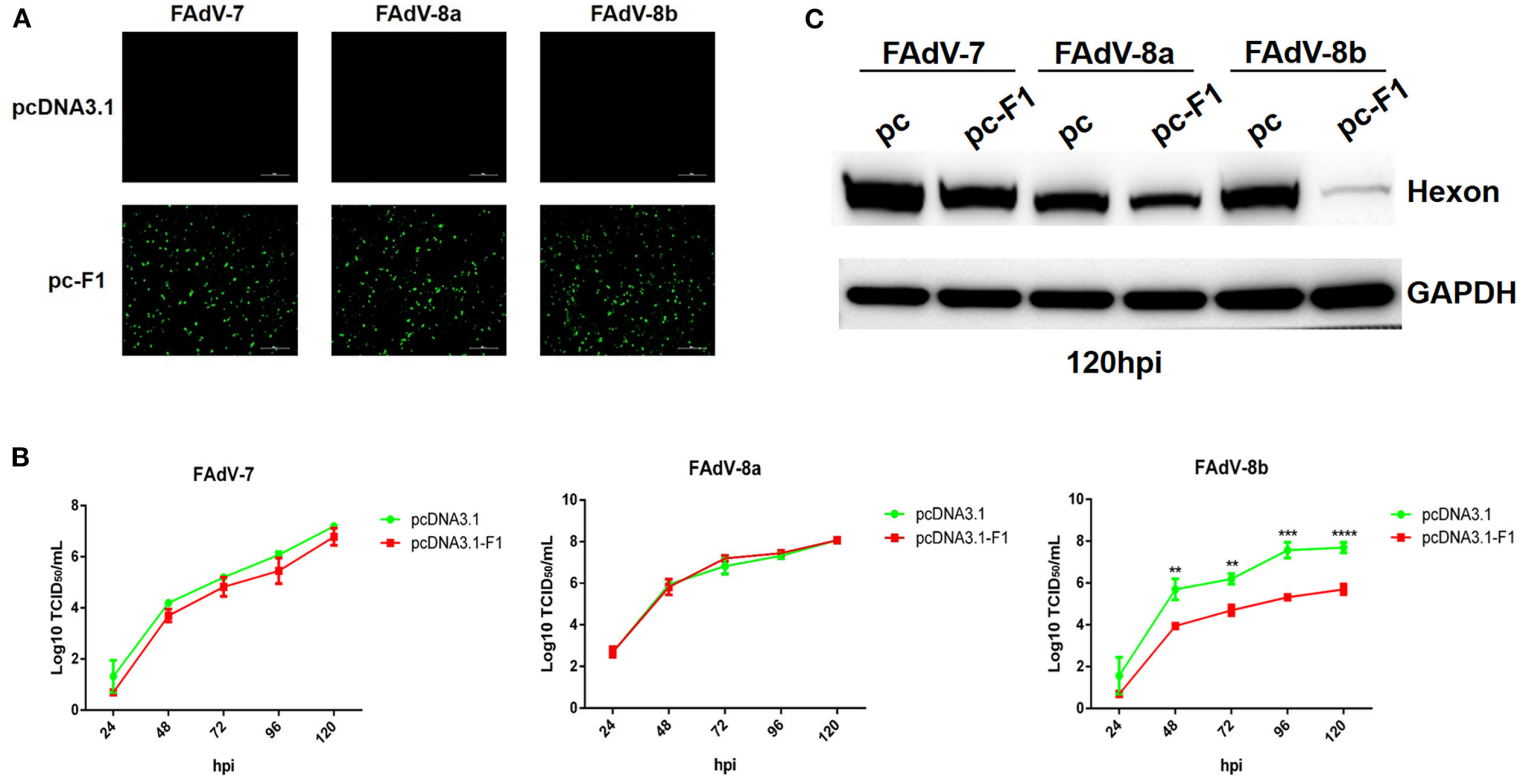
Fiber-1 of FAdV-4 conferred efficient superinfection resistance against FAdV-8b. **(A)** LMH cells transfected with pcDNA3.1-F1 of FAdV-4 were analyzed by IFA using mAb 3B5 against Fiber-1 of FAdV-4. **(B)** Supernatants from the LMH cells infected with FAdV-7, FAdV-8a, and FAdV-8b at different time points, which were transfected with pcDNA3.1-F1 and pcDNA3.1 and titrated with TCID_50_. **(C)** LMH cells infected with FAdV-7, FAdV-8a, and FAdV-8b at 120 hpi, which were transfected with pcDNA3.1 and pcDNA3.1-F1 and analyzed by Western blot using mAb 1B5 against the hexon protein of FAdVs.

Recently, our group and Pan et al. reported that the Fiber-1 of FAdV-4 directly mediated the infection of FAdV-4 *via* the cellular receptor CAR (Pan et al., 2020; Wang et al., 2020). To investigate whether FAdV-8b can infect LMH cells through a similar cell receptor used by FAdV-4, we tested the superinfection resistance assay against FAdV-8b using the Fiber-1 of FAdV-4. In this assay, FAdV-7 and FAdV-8a were used as control viruses. As shown in Figure 2A, similar expression levels of Fiber-1 in the LMH cells transfected with pcDNA3.1-F1 were confirmed in the three infection groups (FAdV-7, FAdV-8a, and FAdV-8b). The superinfection resistance assay revealed that the viral titer of FAdV-8b in the LMH cells transfected with pcDNA3.1-F1 was significantly lower than that of the LMH cells transfected with the control pcDNA3.1 at each time point (Figure 2B). Notably, the viral titer of FAdV-8b in the LMH cells transfected with pcDNA3.1-F1 was 100 times lower than that of the LMH cells transfected with the control pcDNA3.1 at 96 hpi. However, the viral titers of FAdV-7 and FAdV-8a in the cells transfected with pcDNA3.1-F1 were very similar to those of the LMH cells transfected with the control pcDNA3.1 (Figure 2B). Western blot analysis further confirmed the superinfection resistance activity of the Fiber-1 of FAdV-4 against the infection FAdV-8b. As shown in Figure 2C, the abundant hexon protein of FAdV-8b was found in the cells transfected with pcDNA3.1 at 120 hpi, whereas that in cells transfected with pcDNA3.1-F1 was barely detected. Besides, no significant difference was observed in the expression of hexon protein in FAdV-7 and FAdV-8a either transfected with pcDNA3.1-F1 or pcDNA3.1. These data demonstrate that the Fiber-1 protein of FAdV-4 can efficiently inhibit the viral replication of FAdV-8b but not FAdV-7 and FAdV-8a in LMH cells.

### Purified His-Fiber-1 of FAdV-4 efficiently interfered with the infection of FAdV-8b

**Figure 3:**
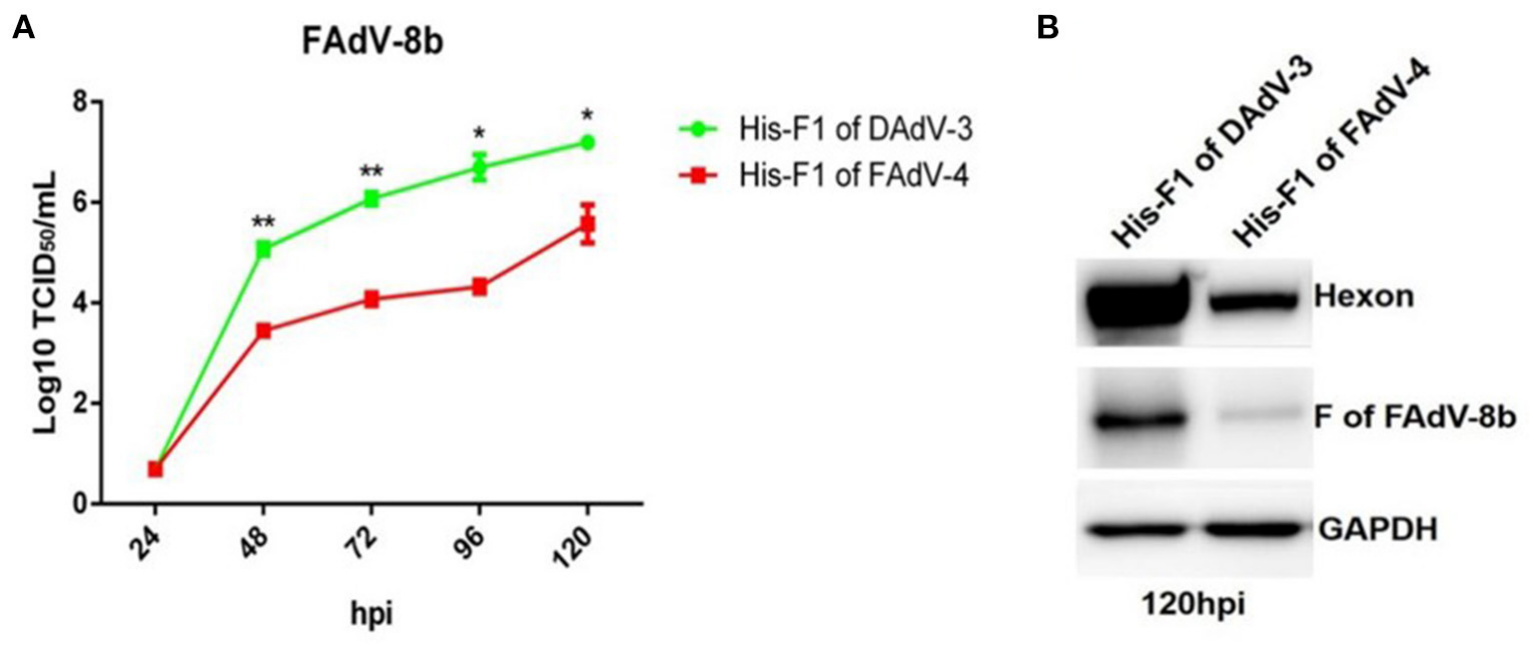
Purified His-Fiber-1 of FAdV-4 efficiently interfered with the infection of FAdV-8b. **(A)** Supernatants from the LMH cells infected with FAdV-8b at different time points, which were treated with purified; His-Fiber-1 of DAdV-3 and His-Fiber-1 of FAdV-4 were titrated with TCID_50_. **(B)** LMH cells infected with FAdV-8b, which were treated with purified; His-Fiber-1 of DAdV-3 and His-Fiber-1 of FAdV-4 were analyzed using mAb 1B5 against the hexon protein of FAdVs and mAb 4D9 against the fiber protein of FAdV-8b by Western blot.

To confirm the superinfection resistance against FAdV-8b by Fiber-1 of FAdV-4, FAdV-8b was first mixed with the purified His-Fiber-1 of FAdV-4 and then inoculated into LMH cells. The His-Fiber-1 of DAdV-3 was used as a control. As shown in Figure 3A, the viral titer of FAdV-8b in the cells incubated with His-Fiber-1 of FAdV-4 was significantly lower than that of cells incubated with His-Fiber-1 of DAdV-3 at each time point. It should be noted that the viral titer of FAdV-8b in the cells incubated with His-Fiber-1 of FAdV-4 was approximately 100 times lower than that of cells incubated with His-Fiber-1 of DAdV-3 at 96 hpi. In addition, Western blot analysis further confirmed the interfering activity of His-Fiber-1 of FAdV-4 against the infection FAdV-8b. The hexon and fiber protein of FAdV-8b in the infected cells treated with His-Fiber-1 of FAdV-4 were significantly lower than those of cells treated with His-Fiber-1 of DAdV-3 (Figure 3B). Together, these data clearly demonstrate that the purified His-Fiber-1 of FAdV-4, but not the purified His-Fiber-1 of DAdV-3, can efficiently interfere with the replication of FAdV-8b in LMH cells.

### Shaft and knob domains of Fiber-1 of FAdV-4 contributed to the superinfection resistance against FAdV-8b

**Figure 4:**
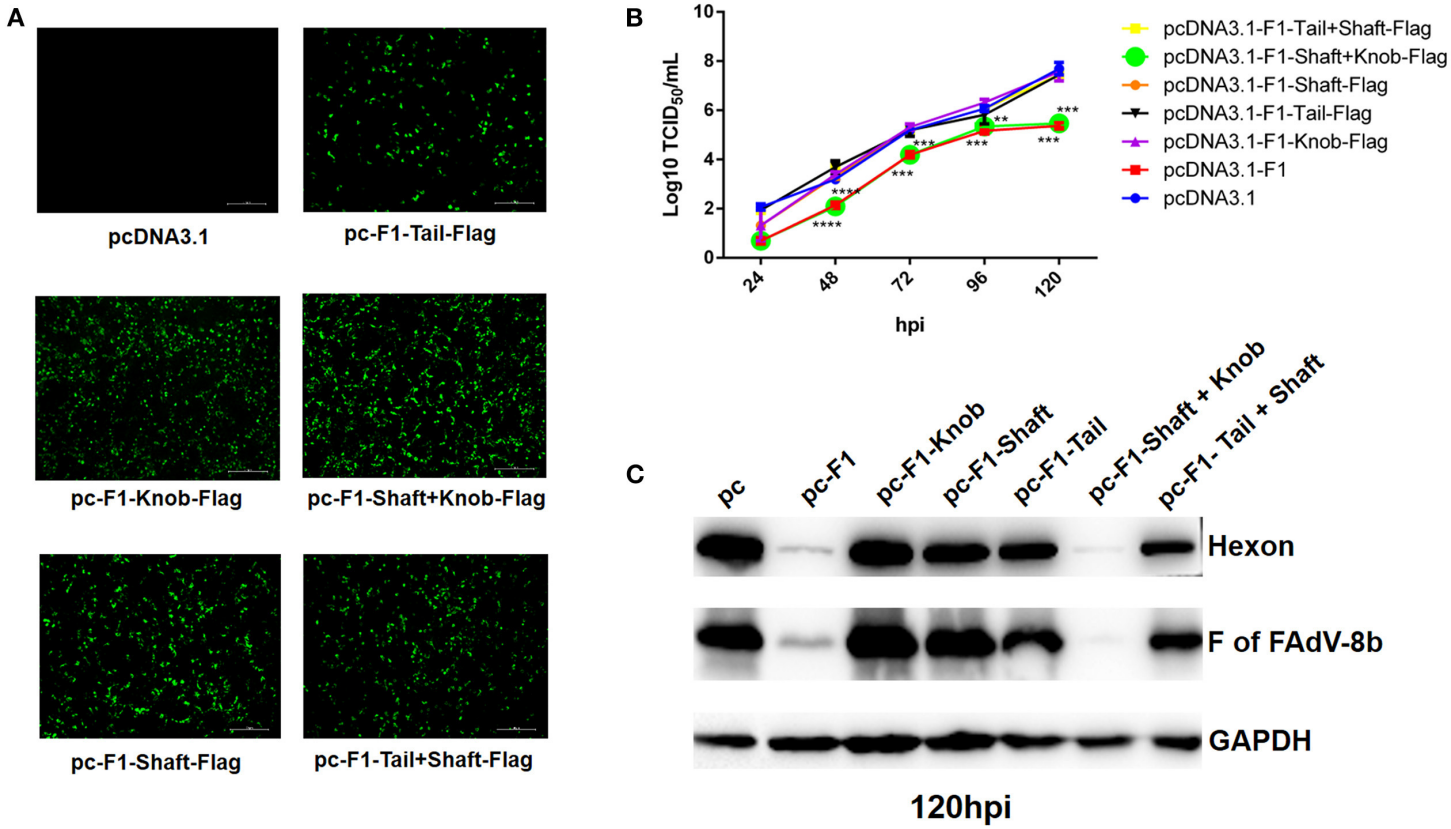
The shaft and knob domains of FAdV-4 Fiber-1 contributed to the superinfection resistance against FAdV-8b. **(A)** Expression of the different truncated Fiber-1 of FAdV-4 in LMH cells; pcDNA3.1-F1-Tail-Flag, pcDNA3.1-F1-Shaft-Flag, pcDNA3.1-F1-Knob-Flag, pcDNA3.1-F1-Tail+Shaft-Flag, pcDNA3.1-F1-Shaft+Knob-Flag, and pcDNA3.1 were transfected into the LMH cells and examined by IFA using mAb against flag. **(B)** Supernatants from the LMH cells infected with FAdV-8b at different time points, which were transfected with pcDNA3.1-F1, pcDNA3.1-F1-Tail-Flag, pcDNA3.1-F1-Shaft-Flag, pcDNA3.1-F1-Knob-Flag, pcDNA3.1-F1-Tail+Shaft-Flag, pcDNA3.1-F1-Shaft+Knob-Flag, and pcDNA3.1 and titrated with TCID_50_. **(C)** LMH cells infected with FAdV-8b at 120 hpi, which were transfected with pcDNA3.1-F1, pcDNA3.1-F1-Tail-Flag, pcDNA3.1-F1-Shaft-Flag, pcDNA3.1-F1-Knob-Flag, pcDNA3.1-F1-Tail+Shaft-Flag, pcDNA3.1-F1-Shaft+Knob-Flag, and pcDNA3.1 and analyzed using mAb 1B5 against the hexon protein of FAdVs and mAb 4D9 against the fiber protein of FAdV-8b by Western blot.

To determine which domain of the Fiber-1 of FAdV-4 contributes to the superinfection resistance against FAdV-8b, a set of Fiber-1 truncations with different domains fused with a flag tag at the C-terminus which were designated as pcDNA3.1-F1-Tail-Flag, pcDNA3.1-F1-Shaft-Flag, pcDNA3.1-F1-Knob-Flag, pcDNA3.1-F1-Tail+Shaft-Flag, and pcDNA3.1-F1-Shaft+Knob-Flag (Fiber-1 truncations), stored in our group, were used. The transfection efficacies and expressions of these Fiber-1 truncations were confirmed by IFA using anti-Flag mAb. As shown in Figure 4A, all of these Fiber-1 truncations were efficiently expressed with similar transfection efficacies. The superinfection resistance assays using these Fiber-1 truncations showed that the viral titers of FAdV-8b in the cells transfected with pcDNA3.1-F1 or pcDNA3.1-F1-Shaft+Knob-Flag were significantly lower than those of cells transfected with pcDNA3.1 at 48, 72, and 120 hpi (Figure 4B). However, the viral titers of FAdV-8b in the cells transfected with other Fiber-1 truncations were very similar to those of cells transfected with pcDNA3.1 at each time point (Figure 4B). Moreover, western blot analysis further confirmed the superinfection resistance activity against the infection FAdV-8b, conferred by shaft and knob domains of the Fiber-1 of FAdV-4. As shown in Figure 4C, the hexon and Fiber protein of FAdV-8b in the cells transfected with pcDNA3.1-F1 or pcDNA3.1-F1-Shaft+Knob-Flag at 120 hpi were barely detected, whereas in contrast, abundant hexon and fiber protein were detected in cells transfected with pcDNA3.1 and other Fiber-1 truncations. Taken together, these data demonstrate that shaft and knob domains of FAdV-4 Fiber-1 are responsible for the superinfection resistance against FAdV-8b.

### Knockout of CAR gene inhibited replication of FAdV-8b in LMH cells

**Figure 5:**
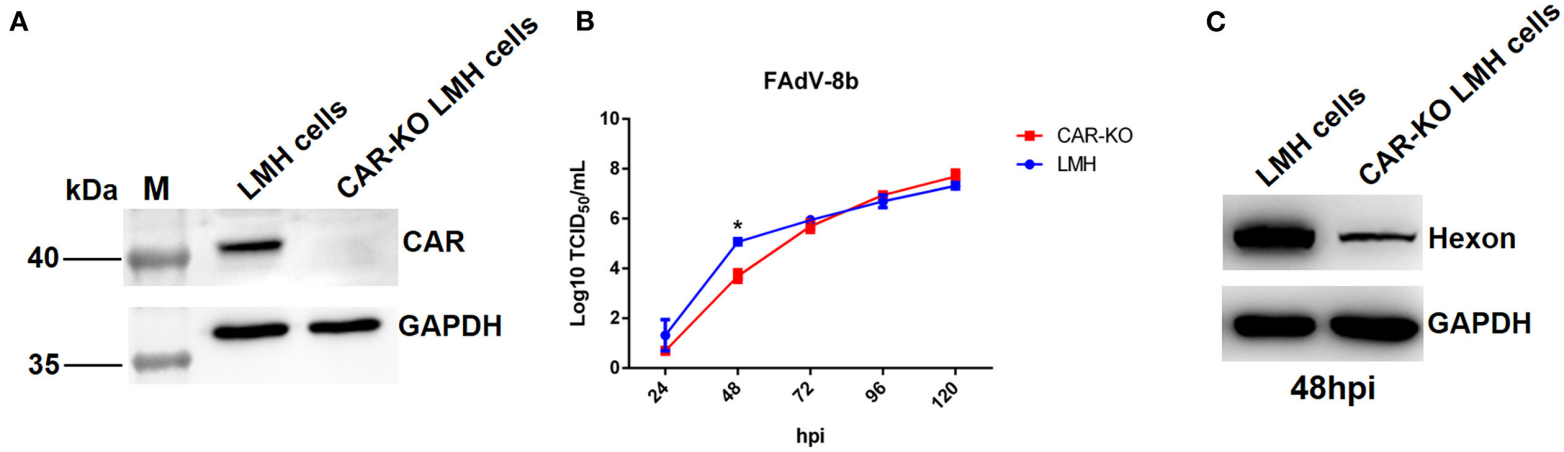
Knockout of the CAR gene in LMH cells affected the replication of FAdV-8b. **(A)** The expression of CAR in CAR-KO LMH cells was examined using polyclonal antibodies against CAR through Western blot. **(B)** Supernatants collected from CAR-KO LMH cells and WT LMH cells infected with FAdV-8b at different time points were titrated with TCID_50_. **(C)** CAR-KO LMH cells and WT LMH cells infected with FAdV-8b at 48 hpi were analyzed using mAb 1B5 against the hexon protein of FAdVs by Western blot.

Previously, Pan et al. identified CAR homology as a cellular receptor for FAdV-4 through its binding with Fiber-1, and our group found that the shaft and knob domains of Fiber-1 mediate the infection of FAdV-4 (Pan et al., 2020; Wang et al., 2020). Hence, to investigate whether CAR plays a vital role in FAdV-8b infection, an LMH cell line with a knockout of CAR was first generated using the CRISPR/Cas9 technique, designated as CAR-KO LMH cells, and further identified by western blot (Figure 5A). As shown in Figure 5B, the viral titers of FAdV-8b in CAR-KO LMH cells were ~50 times lower than that of wild-type (WT) LMH cells at 48 hpi, although no differences were observed at other time points. Western blot analysis further confirmed that knockout of CAR in LMH cells inhibited FAdV-8b replication at 48 hpi. As shown in Figure 5C, the band indicating the FAdV-8b hexon in CAR-KO LMH cells was significantly lower than that of WT LMH cells at 48 hpi. In summary, all these data demonstrate that CAR has a significant effect on the early infection of FAdV-8b *in vitro*.

## Discussion

In recent years, FAdV-8b, as a major causative agent of IBH with worldwide distribution, has caused great economic losses to the poultry industry. However, the molecular mechanism of FAdV-8b infection remains unclear. Numerous studies have shown that fiber proteins of FAdV-4 play critical roles in mediating viral infection and in inducing immune responses (Henry et al., 1994; Ruan et al., 2018). The expression of Fiber-1, but not Fiber-2, could efficiently block the infection of FAdV-4 through superinfection resistance (Wang et al., 2020). In this study, we found that hexon, penton, and fiber proteins of FAdV-8b failed to block the infection of FAdV-8b in LMH cells through superinfection resistance analysis. However, Fiber-1 of FAdV-4 could effectively inhibit the replication of FAdV-8b in LMH cells. Further study revealed that the shaft and knob domains of Fiber-1 of FAdV-4 were responsible for inhibiting the infection and replication of FAdV-8b. Notably, sequence analysis revealed that the fiber of FAdV-8b showed higher identity with the Fiber-2 of FAdV-4, rather than Fiber-1. Previous studies demonstrated that the Fiber-2 of FAdV-4 played a vital role in determining the viral virulence and replication but not in mediating viral infection (Xie et al., 2021; Zhang et al., 2021). Although Fiber-2 shows great promise as a vaccine candidate for FAdV-4, it was not able to induce neutralizing antibodies against FAdV-4 (Schachner et al., 2014). Similarly, chickens vaccinated with the fiber of FAdV-8b could not generate neutralizing antibodies against FAdV-8b (De Luca et al., 2020). This may partially explain why the fiber of FAdV-8b fails to block the infection of FAdV-8b in the superinfection resistance assay. However, the role of the fiber of FAdV-8b in the pathogenesis of FAdV-8b needs to be further elucidated.

Generally, adenovirus infection is initiated by the interaction between a viral fiber and a cellular receptor. CAR is currently the most-studied adenovirus receptor (Stevenson et al., 1995; Zhang and Bergelson, 2005). A recent study revealed that FAdV-4 infection was triggered by Fiber-1 *via* binding with CAR homology (Pan et al., 2020). Although our study showed that the shaft and knob domains of Fiber-1 of FAdV-4 could efficiently block the infection of FAdV-8b through superinfection resistance, the knockout of CAR in LMH cells inhibited the replication of FAdV-8b only at early time points, and the overexpression of CAR did not significantly promote FAdV-8b replication (data not shown), indicating that CAR might not be the key cellular receptor for mediating the infection of FAdV-8b. In addition, although the knob domain was considered critical for adenovirus infection by binding to cellular receptors, the knob alone, without the shaft domain, was not able to inhibit the infection of FAdV-8b in the superinfection resistance assay. This might be related to the functional conformation structure of the knob, which is dependent on the trimerization mediated by the shaft domain of Fiber-1 (Harakuni et al., 2016). Notably, mice polyclonal antibodies against Fiber-1 or the knob domain of Fiber-1 of FAdV-4 could not efficiently block the infection of FAdV-8b (data not shown). Interestingly, sera from chickens infected with FAdV-4, but not vaccinated with inactivated FAdV-4, were able to cross-neutralize FAdV-8b (data not shown). This indicates that the natural exposure of protective epitopes on Fiber-1 of FAdV-4 during infection might play a vital role in mediating a certain cross-protection of FAdV-4 against FAdV-8b.

## Conclusion

This is the first demonstration that Fiber-1 of FAdV-4, instead of the hexon, penton, or fiber of FAdV-8b, can effectively inhibit the infection and replication of FAdV-8b, verified through superinfection resistance assay and interfering analysis, highlighting the novel cellular receptors common for both FAdV-4 and FAdV-8b and the potential cross-protection between FAdV-4 and FAdV-8b. However, the molecular mechanism of the Fiber-1 of FAdV-4 on the inhibition of infection or replication of FAdV-8b requires further investigation.

## Data availability statement

The original contributions presented in the study are included in the article/supplementary material, further inquiries can be directed to the corresponding authors.

## Author contributions

HL, YG, and JY designed the study and analyzed the data. HL, QX, and JY wrote the manuscript. HL, YG, ZX, WW, and ML performed the experiments. TL, ZW, HS, and AQ analyzed the data. JY and QX supervised the experiments and acquired the research funds. All authors contributed to the article and approved the submitted version.
